# Discal Cyst, a Physical Risk: A Case Report on Endoscopic Resection

**DOI:** 10.7759/cureus.32517

**Published:** 2022-12-14

**Authors:** Eng Kee Tan, Mohd Hezery Harun, Noor'Ain Mohd Nasir, Fadzrul Abbas Mohamed Ramlee, Teck Siang Lim, Mohd Nizlan Mohd Nasir

**Affiliations:** 1 Orthopaedic Surgery, Ministry of Health Malaysia, Selangor, MYS; 2 Orthopaedic Surgery, Universiti Putra Malaysia, Serdang, MYS; 3 Pathology, Universiti Putra Malaysia, Serdang, MYS

**Keywords:** endoscopic spine surgery, radiculopathy, back pain, spine, discal cyst

## Abstract

Discal cysts are a rare diagnosis involving the formation of an intraspinal extradural cyst. They are a diagnostic challenge as it is difficult to differentiate discal cysts from other causes of back pain, neurological deficit, and radiculopathy. Due to its rarity, there is a lack of research-based evidence on the optimal management of the discal cyst. This case report aims to increase awareness of this diagnosis and to highlight a possible treatment option for this condition.

## Introduction

Discal cysts are a rare spinal phenomenon where an intraspinal extradural cyst forms, with a connection to its corresponding intervertebral disc [1]. They are uncommon and difficult to clinically differentiate from other causes of back pain, neurological deficit, and radiculopathy. However, with the increasing availability of advanced imaging methods, their diagnosis has become less challenging. Due to their uncommon incidence, the management of discal cysts remains controversial. Deciding on the timing of surgical intervention, if indicated, is somewhat arduous. We report a patient with a discal cyst who underwent an endoscopic excision.

## Case presentation

A 20-year-old male archer presented with a three-month history of lower back pain associated with radicular pain down the left L5 dermatome which was his predominant symptom. He also complained of numbness over the dorsum of his affected foot. There was neither history of preceding trauma, nor was there any history of heavy lifting that precipitated his symptoms. There was no urinary or bowel incontinence. Physical examination revealed Medical Research Council Muscle Power Scale.

Grade 4 of left great toe extension (L5 motor distribution). The sensation was reduced at the L4 to S1 dermatome and the straight leg raise test was positive at 50 degrees. The pain also affected his sports training and performance.

**Figure 1:**
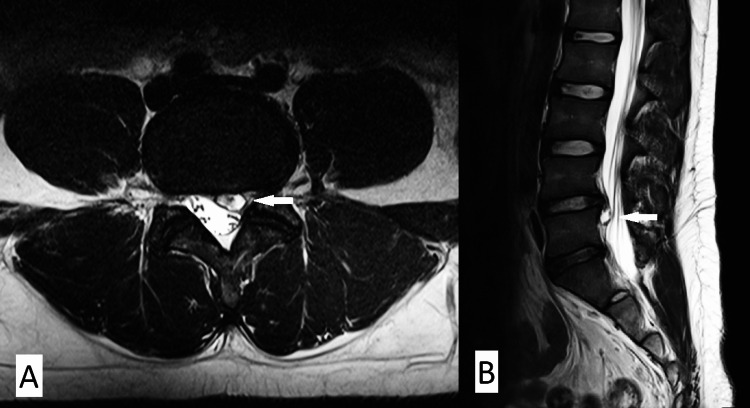
Lumbosacral MRI. Axial (A) and sagittal (B) views demonstrating discal cyst (white arrow)

Lumbosacral MRI revealed a mild diffuse disc bulge at the L4/L5 intervertebral disc. There was also a well-defined lesion at the left paracentral epidural region at the level of L4/L5 measuring 0.7 cm x 0.8 cm x 0.8 cm (AP x W x CC). The lesion was hypointense in T1W and homogenously hyperintense in T2W with surrounding signal void at all sequences. It was adjacent to the left neural foramina, causing stenosis of the spinal canal at the lateral recess region leading to compression of the left L5 traversing nerve roots (Figure 1).

**Figure 2:**
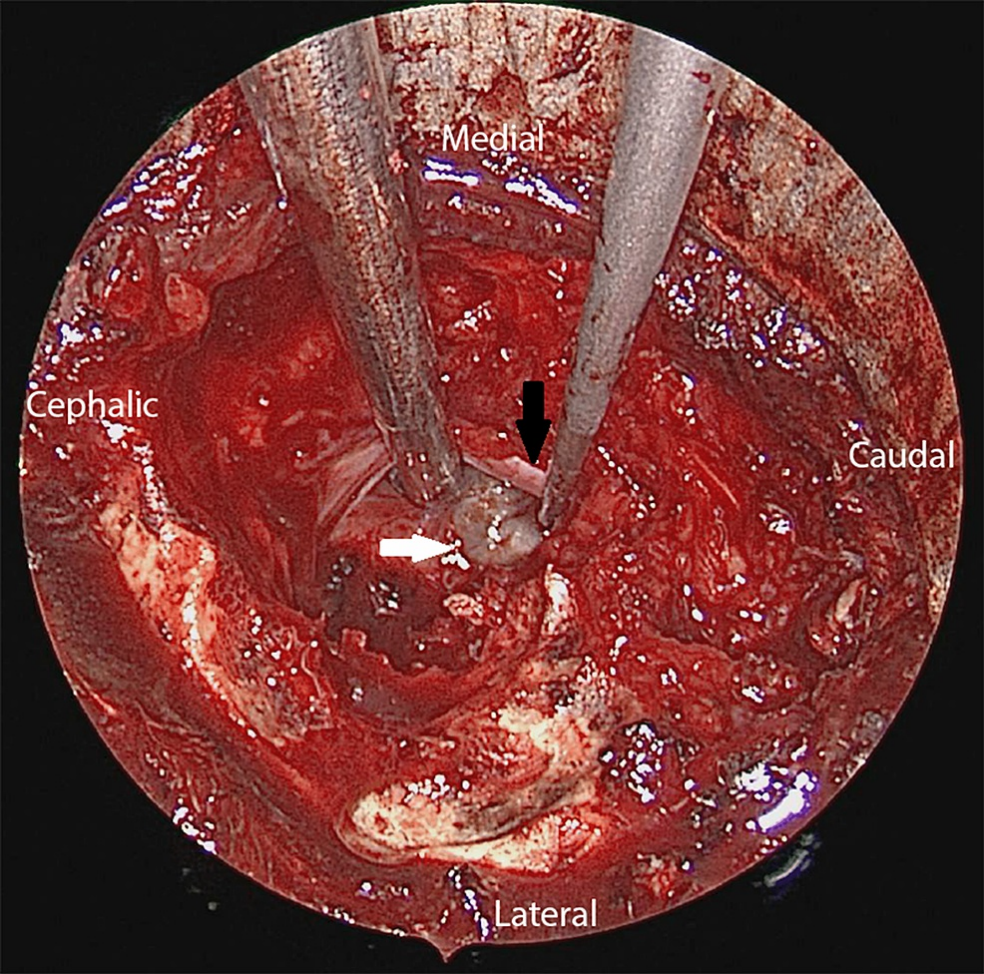
Intraoperative endoscopic image. Discal cyst (white arrow) and the L5 traversing nerve root (black arrow)

An endoscopic microdiscectomy and cystectomy were performed. Intra-operatively, there was an L4/L5 disc bulge with a cystic lesion located just inferior to it compressing the L5 traversing nerve root (Figure 2). It was an encapsulated cyst and upon decompression, it revealed clear fluid content. The stump of the capsule adhered to the L4/L5 annulus fibrosus. There was also a partial tear over the outer region of the annulus. The cyst was completely resected and sent for histopathological examination. The L4/L5 disc was left untouched. The L5 traversing nerve root was well decompressed and noted to be freely mobile with the restoration of blood flow after resection of the cyst.

**Figure 3:**
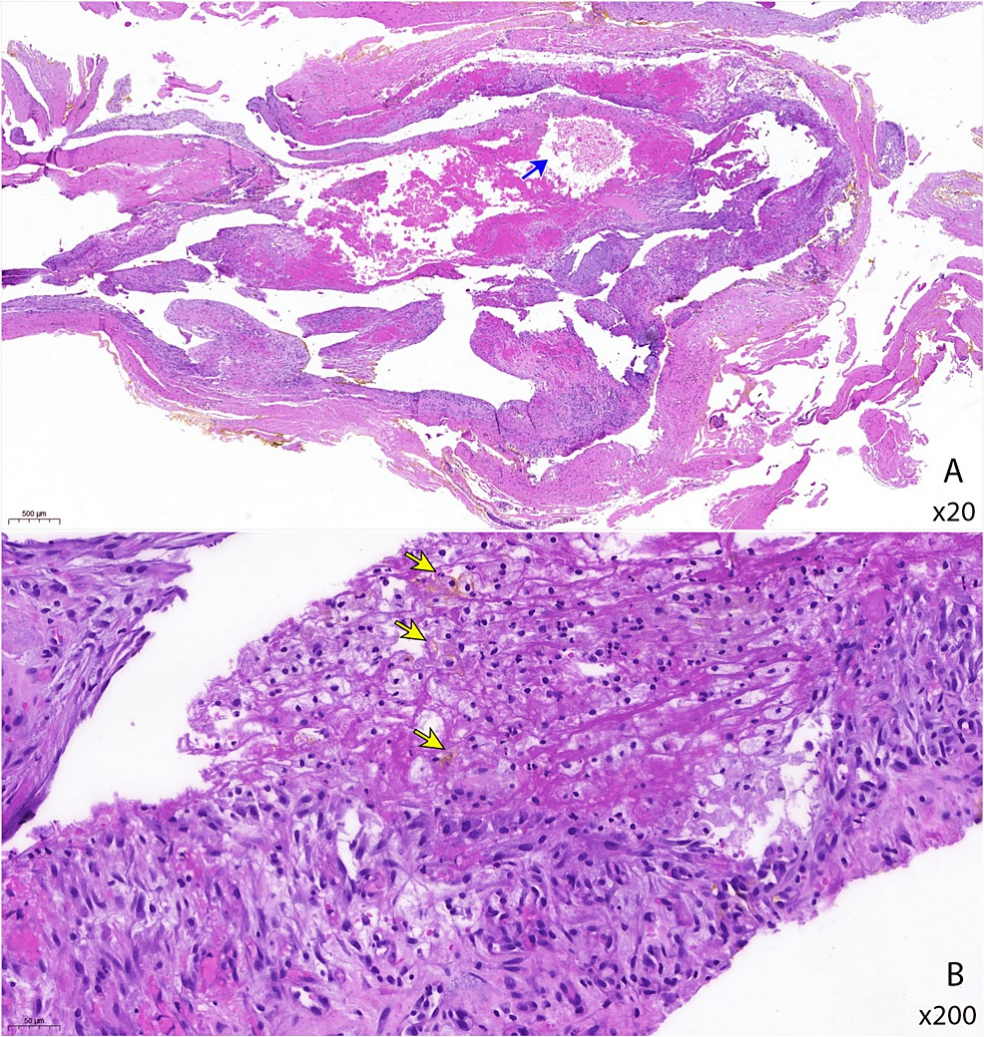
Histopathological image of cyst wall with hematoxylin and eosin stain. The wall is infiltrated by inflammatory cells with areas of hemorrhages (blue arrow) and focal myxoid degeneration are seen at the lower magnification (A). On higher magnification (B), the fibrous wall is infiltrated by mixed inflammatory cell. There is the presence of histiocytes, lymphocytes, neutrophils, and plasma cells infiltrating the cyst wall. Occasional hemosiderin-laden macrophages displaying intracellular brown pigments are seen (yellow arrows) in this photo. Scattered extravasated red blood cells are noted within the interstitium.

Histopathological examination showed the cyst wall was composed of fibrocollagenous tissue that was devoid of the epithelial lining. The wall was infiltrated by mild to moderate amounts of mixed inflammatory cells, such as lymphocytes, histiocytes, neutrophils, foamy macrophages, and plasma cells. Some hemosiderin-laden macrophages and areas of hemorrhage were present. There was no presence of granuloma or malignancy. The case was signed out as an inflamed benign cyst (Figure 3). The patient recovered well after surgery and rehabilitation with no residual neurological deficit.

## Discussion

Discal cysts are an uncommon pathology that can pose a diagnostic challenge to the treating surgeon. Their clinical signs and symptoms often mimic and are indistinguishable from a typical disc herniation presenting with a unilateral nerve root impingement [2-3]. Therefore, it is important to perform an MRI in order to distinguish a discal cyst from other more common diagnoses. In this patient, the lesion was well defined and hyperintense on T2W with surrounding signal void. A contrast medium could be considered for enhancement of the lesion if there is doubt in the diagnosis. These MRI findings are consistent with an article written by Chiba et al. [4].

Discal cysts are most commonly found at the level of L4/L5, the location where there is the largest amount of motion of the lumbar spine. Lee et al. have reported that patients with discal cysts have adjacent disc herniation in more than half the samples. This led the authors to speculate that discal cysts develop from annular fibrosus tears caused by degeneration [5].

While the pathogenesis of the discal disc remains unclear, there have been numerous postulations as to its origins such as hematoma formation deep to the peridural membrane, a pre-existing herniation, or a ganglion cyst originating from the intervertebral disc [1]. In this patient, there was a disc herniation at the level of origin of the discal cyst. There was also no evidence of hemorrhage being the primary cause of the discal cyst as evidenced by both the intra-operative and histopathological examination findings. Due to the lack of epithelial lining in the histopathological sample, the discal cyst in this case report was considered to be a pseudocyst rather than a true cyst. All these indicate a degenerative mechanism in the pathogenesis of the discal cyst.

In the case series written by Chiba et al., only one out of eight patients demonstrated clear serous cystic contents. The remaining seven patients had varying amounts of blood in their cyst contents. This could indicate different underlying causes that lead to the pathogenesis of the discal cyst. However, they also postulated that a discography prior to surgery could have been the cause of the bloody contents in their report [4]. It was also proposed that the hemorrhage could have been caused by an epidural vein rupture caused by mechanical irritation from a herniated disc [5].

Both Chiba et al. and Lee et al. have found that histopathologically, discal cysts consist of fibrous tissue with some degree of myxoid degeneration. They also report hemosiderosis in some specimens, inflammatory cells, and macrophages in some specimens. These findings are consistent with the histopathological examination features in this report [4-5].

Treatment of the discal cyst remains controversial, with varying outcomes favoring different methods of management. Koga et al. have reported a good outcome with treatment using a CT-guided puncture with steroid injection [2]. While this method is minimally invasive, there is a risk of injury to the nerve root if the puncture is not performed accurately [6]. There have also been reports of successful outcomes after non-surgical treatment [7] such as the case in the report by Chou et al. [8]. However, the rate of recurrence for these treatment methods is unknown. In this report, the decision of surgical excision was made due to severe radicular pain associated with weakness that affected his performance as a national athlete.

## Conclusions

The discal cyst remains an elusive diagnosis. With its clinical features mimicking a disc herniation and its underlying pathogenesis unclear, it proves to be a diagnostic challenge, especially to surgeons and physicians without access to more advanced imaging techniques. Treatment remains controversial, as it is difficult to determine the best option due to the rarity of the condition causing a lack of clinical evidence and research opportunities. It is, therefore, important that clinicians be made aware of this diagnosis in order to have a high index of suspicion when treating patients with back pain and associated radiculopathy.
